# The p53 tumor suppressor protein protects against chemotherapeutic stress and apoptosis in human medulloblastoma cells

**DOI:** 10.18632/aging.100831

**Published:** 2015-10-27

**Authors:** Sarah Waye, Aisha Naeem, Muhammad Umer Choudhry, Erika Parasido, Lucas Tricoli, Angiela Sivakumar, John P. Mikhaiel, Venkata Yenugonda, Olga C. Rodriguez, Sana D. Karam, Brian R. Rood, Maria Laura Avantaggiati, Chris Albanese

**Affiliations:** ^1^ Lombardi Comprehensive Cancer Center and Department of Oncology, Georgetown University Medical Center, Washington, DC 20057, USA; ^2^ Department of Radiation Oncology, University of Colorado, Denver, CO 80208, USA; ^3^ Center for Cancer and Immunology Research, Children's National Medical Center, Washington, DC 20057, USA; ^4^ Department of Pathology, Georgetown University Medical Center, Washington, DC 20057, USA

**Keywords:** p53, apoptosis, autophagy, medulloblastoma, Endonuclease G, BIK, p63, p73

## Abstract

Medulloblastoma (MB), a primitive neuroectodermal tumor, is the most common malignant childhood brain tumor and remains incurable in about a third of patients. Currently, survivors carry a significant burden of late treatment effects. The p53 tumor suppressor protein plays a crucial role in influencing cell survival in response to cellular stress and while the p53 pathway is considered a key determinant of anti-tumor responses in many tumors, its role in cell survival in MB is much less well defined. Herein, we report that the experimental drug VMY-1-103 acts through induction of a partial DNA damage-like response as well induction of non-survival autophagy. Surprisingly, the genetic or chemical silencing of p53 significantly enhanced the cytotoxic effects of both VMY and the DNA damaging drug, doxorubicin. The inhibition of p53 in the presence of VMY revealed increased late stage apoptosis, increased DNA fragmentation and increased expression of genes involved in apoptosis, including *CAPN12* and *TRPM8, p63, p73, BIK, EndoG, CIDEB, P27^Kip1^ and P21^cip1^*. These data provide the groundwork for additional studies on VMY as a therapeutic drug and support further investigations into the intriguing possibility that targeting p53 function may be an effective means of enhancing clinical outcomes in MB.

## INTRODUCTION

Medulloblastoma (MB) is a primitive neuroectodermal tumor that arises from granule neuron precursors in the cerebellum or from neural stem cells of the rhombic lip and is the most frequently diagnosed malignant brain tumor in children [1]. Approximately 70% of MB cases occur in children under the age of 10. While less common, MB is also seen in patients between 20 and 44 years of age, with incidences falling off significantly thereafter. A combination of surgery, radiotherapy, and chemotherapy has contributed to improved treatment outcomes, resulting in a 70-80% five-year disease-free patients with medulloblastoma remain significant and recurrence is frequently observed. As with many malignancies, disease recurrence is nearly always fatal, and late mortality remains a serious health issue in long-term MB survivors [2]. Moreover, current therapies result in significant negative impacts on neurological, cognitive and social development, especially in the youngest affected children. Significant efforts are therefore underway to develop more effective and less toxic MB treatments.

The efficacy of many anti-tumor agents relies on their ability to trigger the tumor suppressive activities of p53, which leads to the induction of cell death, frequently via cellular pathways of apoptosis, senescence or mitotic catastrophe. While the activity of the p53 tumor suppressor protein is highly complex [3], its expression is induced by a broad array of cell stressors including DNA-damaging chemotherapeutic drugs and can be an excellent target for therapeutic intervention ([4], see also [3]). Impairment of *p53* signaling by gene mutation or gene silencing/loss has been shown to contribute to the induction, progression and/or recurrence of many tumor types and can confer resistance to tumor therapy.

p53 plays unique roles in neural development. For example, p53 has been directly implicated in neurogenesis as well as in neural stem cell self-renewal, neurite outgrowth and axonal regeneration (reviewed in [5]), and acetylation of p53 is required for the induction of neurite outgrowth [6]. Despite this knowledge and that related to the role of p53 in many malignancies, the function of p53 in MB remains under-explored. For example, unlike lung, pancreas and bladder cancers, only a minority of primary MB patients present with p53 mutation or loss, with reported frequencies between 7% [7] and 15% [8]. Interestingly, while the frequency of p53 mutations increases upon recurrence, the percentage of cells with nuclear p53 also increases, rising from 26% at diagnosis to 33% at relapse [8], suggesting that certain mechanisms underlying p53 function may still be intact. Importantly, the MAGIC consortium identified chromosome 17 deletions, where the *p53* locus is located, to be associated with chromothripsis (chromosomal fragmentation) in Group 3 MB [9], while reduced expression of p53 was seen in Group 4 MB [10]. Collectively, these findings highlight the complex and poorly defined role for p53 in human MB, and support the need for mechanistic studies into p53 activity as a possible therapeutic effector protein.

The *in vitro* [11-13] and *in vivo* [14] anti-tumor activities of an experimental CDK inhibitor, VMY-1-103 (VMY), have previously been described by us in both prostate and other solid tumors [11, 13, 15] and in MB [12, 14]. Our previous MB studies established that the extrinsic apoptotic pathway was induced by VMY, as was mitotic catastrophe in a subset of the cells [12]. In the present study, we sought to further define the molecular and genetic mechanisms by which VMY induces MB cell death. Herein, we show in both p53-wild type (D556) and p53-mutant (DAOY) MB cells lines that treatment with VMY resulted in the translocation of p53 into the nucleus, an induction of γH2AX, a decrease in MDM2 protein levels and activation of non-survival macro-autophagy. Interestingly, suppression of p53 function via shRNA knockdown or treatment with the p53 inhibitory compound Pifithrin-α (Pif) [16] resulted in significant increases in cell death following treatment with either VMY or doxorubicin. Gene expression analyses performed on D556 cells treated with VMY and Pif versus VMY alone revealed a significant increase in genes associated with apoptosis and necrosis, including the calcium pathway signaling genes *CAPN12 and TRPM8* suggesting alterations in intracellular calcium signaling may play a role in enhancing cell death. In addition, *p63* and its transcriptional target the proapoptotic gene *BIK* were induced, as were *p73* and its target, the caspase-independent intranucleosomal DNase, *Endonuclease-G* (Endo-G) [17].

Given the difficulties in effectively treating MB, especially recurrent disease, targeting p53 in combination with chemotherapy potentially represents a new treatment strategy for medulloblastoma.

## RESULTS

### Treatment of MB cells induces a durable cytotoxic effect

**Figure 1:**
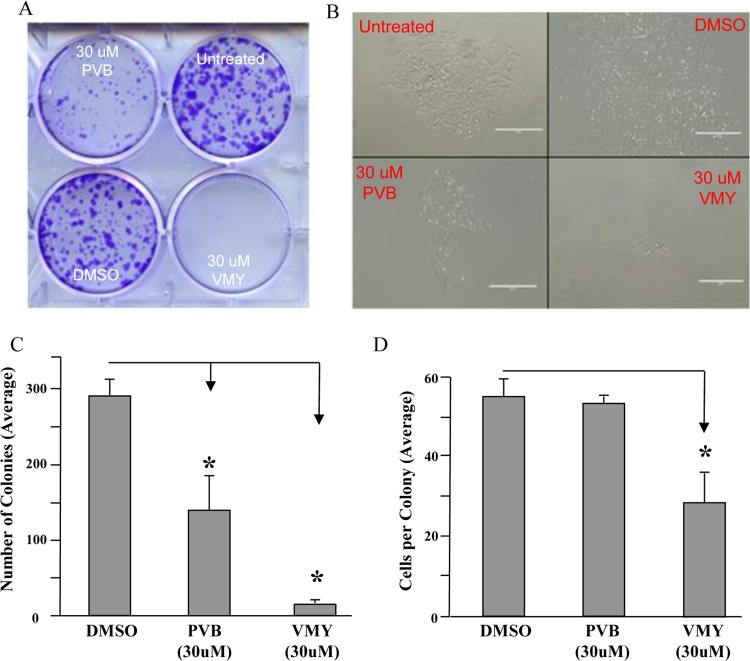
VMY induced cell death The durability of effects of VMY on cell viability was determined via colony forming assays. D556 cells were treated with DMSO, PVB or VMY for 18 hrs. Fresh media was added and the cells cultured for an additional 3-5 days. (**A**) Cells stained with crystal violet. (**B**) Colonies as visualized by microscopy. (**C**) Quantification of colony number. (**D**) Quantification of cells per colony. The data are shown as average + standard deviation. PVB; purvalanol B, *; p<0.05.

We have previously reported that VMY induces MB cell death [12, 14]. To test whether VMY's antiproliferative effects were sustained after removal of the compound, colony forming assays were performed. D556 cells were treated with VMY or its parent compound purvalanol B (PVB) for 18 hrs, at which point the media was changed and the cells were allowed to recover in the absence of the drugs until the control plate reached 80% confluency (approximately 3-5 days). VMY treatment resulted in a significant reduction in both the number of colonies (Fig 1A, B, C) as well as the number of cells per colony (Fig 1D) versus either DMSO- or PVB-treated D556 cells, which express wild type p53. The DNA damaging drug, doxorubicin (1uM), effectively killed all cells (not shown).

### VMY induces a partial DNA damage-like response in DAOY and D556 MB cell lines

**Figure 2:**
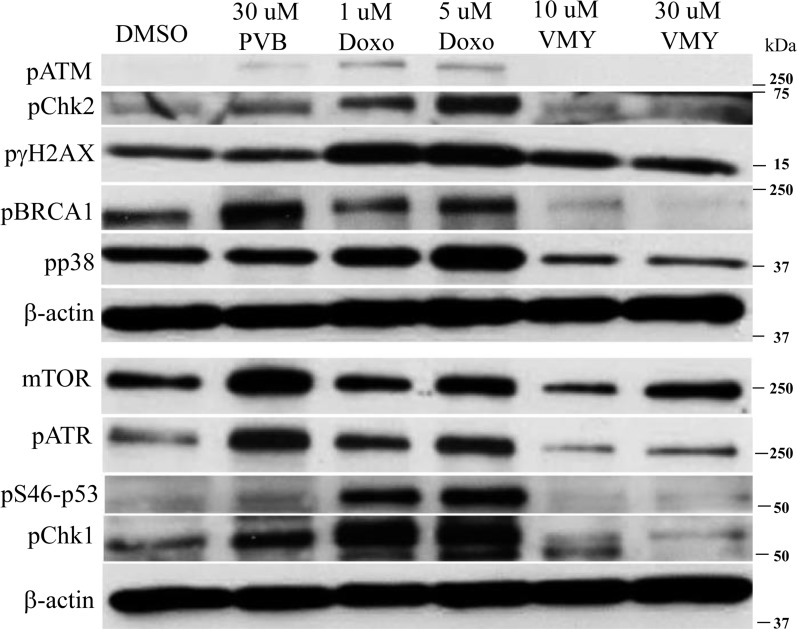
Effects of VMY on stress related proteins DAOY cells were treated for 18 hrs with DMSO, PVB, VMY or doxorubicin at the concentrations listed and immunoblotting was performed for the proteins shown. β-actin was used as a loading control. PVB; purvalanol B, Doxo; doxorubicin.

**Figure 3:**
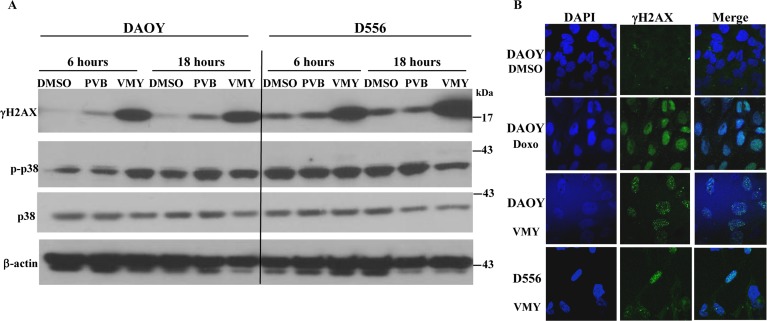
Effects of VMY on stress related proteins in MB cells DAOY and D556 cells were treated with PVB or VMY. (**A**) Immunoblotting was performed for total and phosphorylated p38 and phosphorylated γ-H2AX following treatment for 6 or 18 hrs. (**B**) Immunofluorescence microscopy for γ-H2AX was performed on DAOY cells treated with 1 uM doxorubicin for 18 hrs and DAOY and D556 cells treated with 10 uM VMY for 18 hrs. DAPI was used to stain the nuclei. PVB; purvalanol B, Doxo; doxorubicin.

Our previous studies established that the induction of cell death in MB cells occurred, at least in part, through the extrinsic apoptotic pathway and mitotic disruption [12, 14]. To further investigate the mechanisms by which VMY impacts cell survival, we interrogated proteins involved in DNA damage response and stress signaling. Time course studies of VMY treatment were performed first in DAOY cells, which express mutant p53 (p53^C252F^). Doxorubicin was used as a positive control for induction of a DNA damage response [18], and PVB was also tested. Compared to DMSO control, treatment with doxorubicin for 18 hours increased the levels of phosphorylated isoforms of ATM, Chk2, γH2AX, BRCA1 and p38 (Fig. 2) as well as ATR, pS46-p53 and Chk1 (Fig 2). A modest increase in mTOR was also noted. In contrast, the levels of all of these proteins, with the exception of p-γH2AX (Fig 2) and to a lesser extent mTOR, were reduced following treatment with VMY. Interestingly, PVB behaved in a manner similar to doxorubicin despite the fact that PVB is an inefficient inhibitor of MB cell proliferation [12]. In contrast to DAOY cells, the levels of total- and phospho- p38 remained relatively constant in D556 cells and phospho-p38 decreased slightly following 18 hrs of VMY treatment (Fig 3A), however sustained induction of γH2AX was confirmed by western blot and by immunofluorescence in both DAOY and D556 cells (Fig 3A, B).

### VMY induces autophagy in MB cells

**Figure 4:**
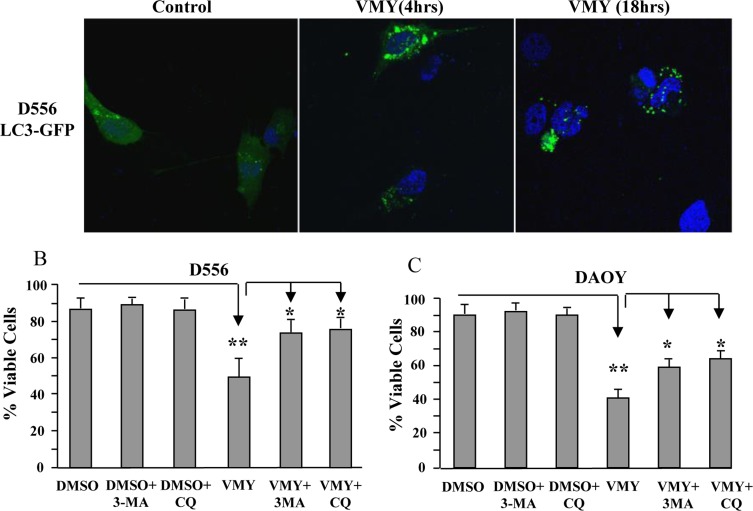
Induction of Autophagy by VMY reduces cell viability (**A**) D556 cells, transfected with LC3-GFP, were treated with DMSO or VMY for 4 and 18 hrs. Autophagic LC3-GFP puncta were visualized by fluorescence microscopy. Cell nuclei were stained with DAPI. (**B**) D556 and (**C**) DAOY cells were treated with VMY for 18hrs in the presence or absence of 5 uM 3-MA (an inhibitor of early autophagy) or 50 uM chloroquine (an inhibitor of acidification of lysosomes and autophagosomes), and trypan blue viability assays were performed to establish cell viability. The data are shown as the average + standard deviation of N=3 separate experiments. *; p<0.05, **; p<0.01, 3-MA; 3-methyladenine, CQ; chloroquine.

VMY has the ability to block proliferation in prostate cancer cells in part through the induction of catastrophic autophagy [15]. During autophagy, LC3-I (microtubule-associated protein 1 light chain 3) becomes lipidated by the class III phosphoinositide 3-kinase, Vps34, and relocalizes from the microtubules to autophagosomal membranes (reviewed in Kang, et al. [19]). We therefore studied the pattern of subcellular localization of LC3-I in MB cells. D556 cells were transiently transfected with an LC3-GFP expression vector and subjected to fluorescence microscopy as previously described [15]. VMY treatment induced LC3-GFP relocalization and concentration into prototypical autophagic puncta (Fig 4A) with an average of 6 puncta per VMY-treated, LC3-GFP positive cell at 4 hours and 7.8 puncta per cell at 18 hrs, versus an average of 2.3 puncta per cell in control cells (Fig 4A). Our previous data established that inhibition of autophagy protected against VMY-induced cell death in prostate cancer cells [15]. We therefore investigated whether inhibitors of early (3-methyladenine, 3-MA) or late (chloroquine, CQ) autophagy influenced cell survival. Using D556 and DAOY cells, trypan blue dye exclusion assays established that neither 3-MA nor CQ influenced survival in control cells, however significant increases (p<0.05, N=3 separate experiments) in cell viability were seen in both cell lines when treated with VMY and the inhibitors (Fig 4B, C).

### Regulation of p53 activity is similar in DAOY and D556 MB cell lines

Our earlier investigations into the mechanisms by which VMY reduced overall cell survival in solid tumors clearly established a role for wild type p53 in inducing cell death through both apoptosis and catastrophic autophagy. For example, in adenocarcinoma cell lines with wild type p53, VMY caused a rapid induction of p53 protein levels whereas p53 levels remained constant in cells harboring p53 mutations [15]. Furthermore, the loss of p53 function via deletion, mutation or genetic silencing resulted in a complete loss of VMY-induced cytotoxicity in a variety of cancers, including prostate, breast and pancreas, while re-expression of wild type p53 in PC3 cells or treatment of DU145 cells with PRIMA1 restored VMY-induced autophagy and cell death [11, 15].

**Figure 5:**
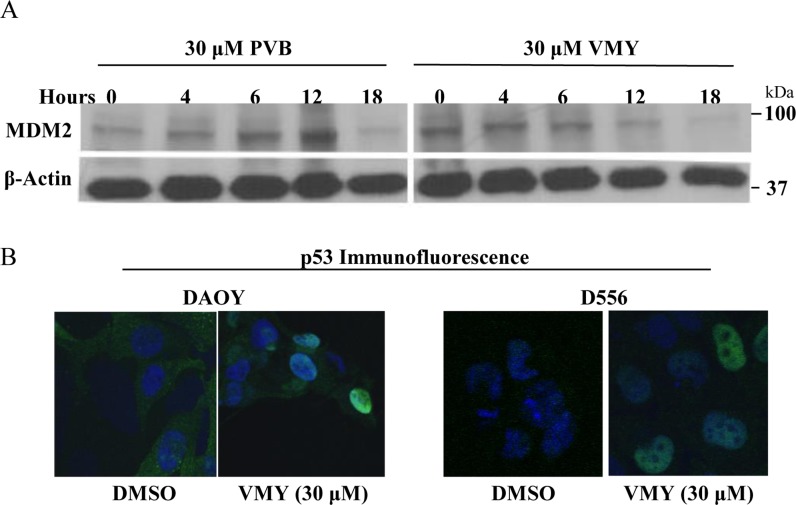
VMY alters the subcellular localization of p53 (**A**) Immunoblotting for MDM2 following exposure of D556 cells to PVB and VMY for the times indicated. β-actin was used as a loading control. (**B**) Immunofluorescence microscopy for p53 subcelluar localization was performed on DAOY (left panels) and D556 (right panels). DAPI was used to stain the nuclei. PVB; purvalanol B.

We therefore next investigated the effects of VMY on p53 expression in DAOY (p53 C242F mutant [20]) and D556 cells (p53 wild type). Unlike our previous findings in adenocarcinoma cells, p53 levels were high in both cell lines and were not affected by treatment with VMY (Supplemental Fig. 1). Similar results were seen with PVB (Supplemental Fig. 1). The levels of the p53-regulatory protein MDM2 were decreased in both cell lines (Fig 5A) and immunofluorescence microscopy demonstrated that p53 shifted from diffusely cytoplasmic with some nuclear positivity in control cells to predominantly nuclear in both cell lines following VMY treatment (Fig 5B). As both the wild type and mutant p53 proteins localize to the nucleus following exposure to VMY, these data suggest that both proteins may retain some functional activity.

### The role of p53 in inducing cell death

**Figure 6:**
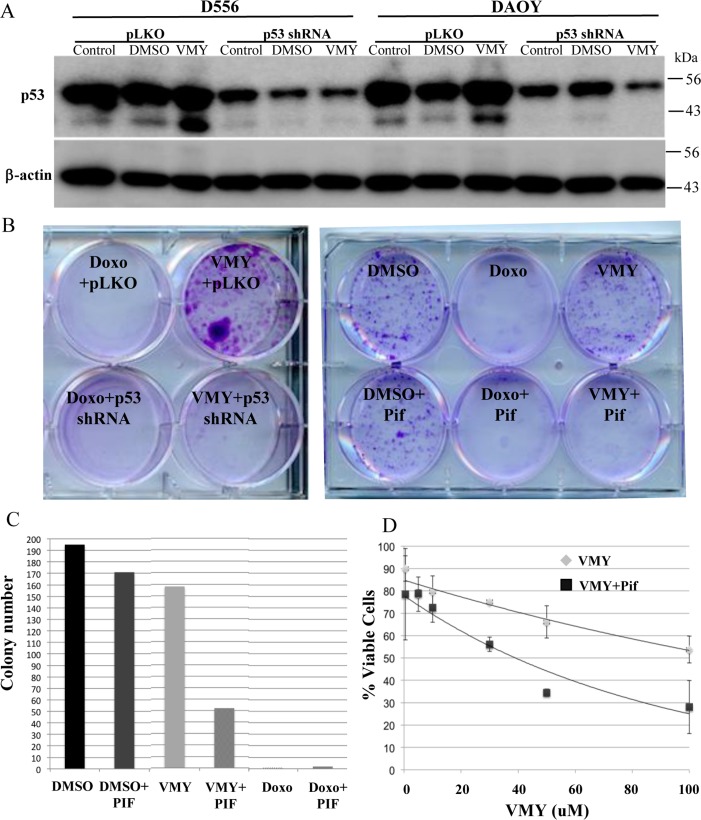
Effects of silencing of p53 on cell survival (**A**) Genetic silencing of p53. D556 and DAOY cells were infected with p53siRNA or pLKO lentivirus'. The cells were left untreated or exposed for 18 hrs to DMSO or 30uM VMY as indicated, and western blots for p53 and β-actin were run. (**B**) The effects of p53shRNA knockdown (left) and Pifithrin (Pif, right) on cell viability were determined via colony forming assays. D556 cells were treated with DMSO, doxorubicin or VMY for 18 hrs. Fresh media was added and the cells cultured for an addition 3-5 days, followed by staining with crystal violet. (**C**) Quantification of the number of colonies in (**B**). (**D**) Dose response curves of D556 cells treated with VMY at the concentrations shown in the presence and absence of Pifithrin. The data are shown as average + standard deviation of N=3 separate experiments.

To determine the role of p53 in regulating MB cell survival in the presence of VMY, p53 was genetically silenced with the previously validated p53 shRNA [15] or chemically inhibited by the p53-inhibitory compound, Pifithrin-α (Pif), which we have used in previous experiments to investigate p53's role in regulating autophagy [16]. The silencing of p53 by shRNA resulted in up to a 68% decrease in p53 protein levels versus pLKO control across all treatment groups in both D556 and DAOY cells (Fig 6A). Surprisingly, both the genetic and chemical silencing of p53 led to significant increases in cell death by VMY as measured by colony forming assay (Fig 6B). Equally surprising was the observation that the loss of p53 failed to protect against cell death by doxorubicin (Fig 6B, C). Dose escalation experiments performed in D556 cells in the presence and absence of Pif established that the heightened chemosensitivity was consistent across a broad range of concentrations (Fig 6D). In addition, experiments performed in DAOY showed that cell-survival declined by 33 percent in VMY-treated cells with p53 shRNA knockdown compared to VMY-treated pLKO control cells (Sup Fig S2).

### Loss of p53 in the presence of VMY alters calcium, p63 and p73 signaling pathways

**Figure 7:**
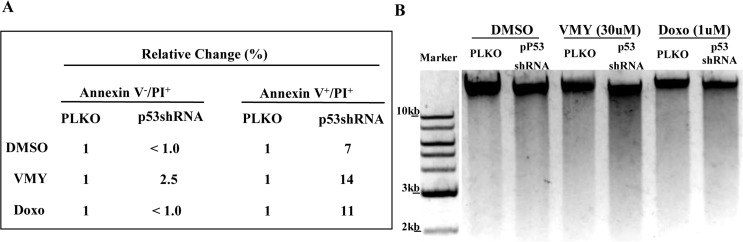
Effects of p53 knockdown on apoptosis and DNA fragmentation in D556 cells (**A**) The proportion of cells undergoing apoptotic cell death as a result of p53 shRNA knockdown in D556 cells treated for 18 hrs with DMSO, VMY (30uM) or doxorubicin (1uM) as assessed using annexin V and propidium iodide (PI) staining and measured by flow cytometry. Data are shown as percent change in staining versus pLKO-control infected cells. (**B**) D556 cells were infected with pLKO or p53shRNA and treated with DMSO, VMY or doxorubicin for 18hrs. DNA fragmentation of nuclear DNA was assessed by ethidium bromide-agarose gel electrophoresis.

In order to more completely define the mechanism underlying the paradoxical effect of p53 silencing, RNAseq next generation sequencing was performed on D556 cells treated with VMY in the presence or absence of Pif. RNA sequence analysis revealed an increase in expression of *calpain 12* in the VMY/Pif treated cells vs. VMY/DMSO control cells (Table 1). In addition, elevated expression of the transient receptor potential channel subfamily (*TRPM8*) gene was seen (Table 1), collectively suggesting that intracellular calcium signaling pathways were affected by p53 silencing. Dysregulation of the calcium signaling pathway downstream of stressors such as excitotoxicity can lead to necrotic cell death in neurons (reviewed in [21, 22]), with one of the hallmarks of necrosis being Endo G induction and intranucleosomal DNA cleavage [22]. As both the pro-apoptosis regulatory genes p63 and p73 were induced by p53 silencing, as were possible downstream targets including Endo-G [23], the proapoptotic BH3-protein, BIK (Bcl-2-interacting killer) and CIDEB (cell death-inducing DFFA-like effector B), we assessed levels of late stage apoptosis and necrosis by flow cytometry, by gating for annexin V-positive/propidium iodide (PI)-positive cells. D556 cells were infected with either pLKO or p53shRNA as described above and treated for 18-hours with DMSO, 30uM VMY or 1uM doxorubicin, after which they were analyzed by flow cytometry as previously described [15]. While the annexin^−^/PI^+^ fraction of cells was unaffected, the silencing of p53 increased the proportion of annexin V^+^/PI^+^ cells following exposure to VMY or doxorubicin (Fig 7A). Finally, similar effects were seen using agarose gel electrophoresis assays where 18-hour treatment with VMY or doxorubicin plus p53shRNA resulted in enhanced DNA degradation, indicative of necrosis and apoptosis (Fig 7B).

**Table 1:** Top 25 genes altered in the presence of Pifithrin α plus VMY vs. VMY alone

Pif + VMY *vs*. VMY			
Up-regulated	Fold	Down-regulated	Fold
EndoG	8.77809E+30	FIGF	−6.10476E+11
CIDEB	1.9687E+12	ANGPT4	−6.017072518
PRSS54	1299.03	GSK3A	−5.35
BIK	19.44	INCA1	−5.18
MAP3K9	18.19	TNFSF14	−4.01
ERBB3	15.17	GDF15	−4.01
BRAT1	7.48	HGF	−3.89
CISH	6.73	BIRC3	−3.41
FADD	6.73	LIF	−2.85
TP63	6.63	NTF3	−2.77
CBX6	6.23	DRD2	−2.75
SRC	5.98	SNCG	−2.67
CBX7	5.98	MAGEA9	−2.67
HDAC4	5.24	ZNF385D	−2.67
RASSF4	4.49	CRIP3	−2.67
TRPM8	4.49	TNFSF15	−2.45
ERBB2	4.06	NAP1L6	−2.45
AKT1	3.84	TENC1	−2.33
UNC5B	3.83	NRCAM	−2.23
TNFRSF10D	3.74	DNAJB7	−2.21
NLRP12-14	3.74	MAGEB2	−2.19
TP73	3.74	PPAPDC2	−2.14
ARHGEF18	3.55	PRSS12	−2.14
TNFRSF25	3.49	CFLAR	−2.07
FASTK	3.48	GADD45A	−2.07

Taken together, these experiments show that p53 protects against drug-induced cell death in medulloblastoma cells and its genetic- or chemical-suppression results in a significant increase in cell sensitivity to VMY and doxorubicin, an experimental and a clinical drug, respectively.

## DISCUSSION

Necrosis, apoptosis and autophagy are activated under a variety of cell stress conditions (see references [24, 25] among others), however, little is known about how these complex and partially overlapping mechanisms are induced in medulloblastoma cells. In addition, to date, there have been few publications exploring the effects in medulloblastoma cells of the synthetic modulation of p53 activity during exposure to chemotherapeutic drugs.

We have recently shown in prostate cancer cell lines as well as in primary prostate cancer cells established using our conditional cell reprogramming approach [26, 27], that the induction of p53 by VMY was a prerequisite for inducing both autophagy and apoptosis, and that silencing p53 effectively blocked cell death [15]. Additionally, our earlier studies on VMY's effects on MB established that this experimental drug induced apoptosis and mitotic catastrophe *in vitro* [12]. Furthermore, while our *in vivo* studies showed that 20 mg/kg of VMY administered three times per week for more than four weeks was well tolerated and was effective at treating a mouse model of SHH-driven medulloblastoma [14], a detailed investigation into the mechanism of VMY-induced cell death, and the role that p53 may play had not been explored. We now show that in MB cells, VMY induces the relocalization of p53 into the nucleus, an accumulation of γH2AX, a decrease in MDM2 protein levels and activation of non-survival macro-autophagy. Since the protein levels of key stressrelated proteins were reduced by VMY, the possibility existed that components of the CAP-dependent protein translation pathway may be inhibited by VMY. MNK1 is a target of p38 and MAPK and acts to increase CAPdependent translation through the phosphorylation of the elongation factor eIF4E [28]. 4E-BP1 is a negative regulator of translation and phosphorylation of 4E-BP1 by mTOR inhibits its repressor function. Thus, if VMY negatively regulated CAP-dependent translation, the phosphorylation levels of 4E-BP1 and pMNK1 would be expected to reduce, however VMY increased the levels of these proteins in both D556 and DAOY cells (S.W and C.A, unpublished data). Interestingly, rather than protecting against chemotherapeutic cell killing, the suppression of p53 through shRNA knoc kdown or chemical inhibition by Pifithrin-α resulted in a significant increase in cell death by either VMY or doxorubicin, suggesting that p53 acts as a chemoprotective protein in these primitive neuroectoderm-derived cancer cells.

Regarding its function in the neuroectoderm, p53 performs roles different to those found in other tissues. In the past decade a role for p53 has emerged in neuronal differentiation, axon guidance, neurite outgrowth and axonal regeneration [29, 30]. Analysis of p53-dependent transcriptional activation in normal development *in vivo* by using a lacZ reporter gene under the control of a p53-responsive promoter showed that p53 activity was maximal during neuronal differentiation and clustered in areas that showed little correlation with the apoptosis normally ongoing in the developing nervous system [31, 32]. Furthermore, other studies have shown that approximately one quarter of p53-null mice developed exencephaly due to cellular overgrowth, rather than decreased apoptosis [33, 34].

The dependence of neurite outgrowth and elongation on p53 has also been shown in the developing cerebellum. Gaub et al., 2010 showed that acetylated p53 is required for neurite outgrowth in cerebellar granule cell progenitors. Conversely, the loss of the function acetyl p53 mutant (K-R) inhibits physiological neurite outgrowth in those cells [35]. In cultured rat cerebellar granule cells, Maruoka et al., 2011, showed a p53-mediated neuroprotective effect against glutathione depletion-induced oxidative stress [36].

Further validation of the role for p53 in neurite outgrowth and neuronal differentiation and maturation comes from studies establishing p53 as a downstream target of neurotrophic receptors. Loss of function experiments of p53 via either gene silencing or dominant negative p53 proteins lacking transactivation capacity have been shown to block NGF-dependent neurite outgrowth and differentiation in PC-12 cells [6, 37]. Another neurotrophic factor, BDNF, has also been shown to stimulate p53 phosphorylation and transcriptional activation in primary cortical neurons [30]. Activation of signaling molecules downstream of NGF or BDNF that are known to induce p53 posttranslational modifications and enhance its transcriptional activity has been reported, including ERK1 and ERK2, p38MAPK, JNK1-2 (c-Jun Nterminal kinases 1-2), cytoskeleton remodeling genes, such as GAP-43, the actin-binding protein Coronin 1b and the RAS family member Rab13 [6, 38].

Unresolved however is an actual role for p53 in the biology of human MB. Frequencies of *p53* mutations are low in primary MB but Increase significantly in recurrences, and mutant p53 proteins and Myc may collaborate to drive aggressive disease [8]. Additionally, modifications of p53 function are required in *Myc*-[39, 40] but not *Smoothened*-based mouse models to drive MB. The genetic silencing of *p53* in mice with conditional deletion of the *BRCA2-interacting protein* (*BCCIP*) gene also resulted in MB [41]; however the resulting tumor formation was predicated upon the loss of the BCCIP knockdown cassette, which restored BCCIP expression in the neuroectoderm, supporting a role for p53 in neuronal genomic stability. Interestingly, p53 expression levels are lower in group 4 MB, due to the iso-dicentric (17)(p11.2) recombination events frequently seen in this group [10]. However, neither the levels of p53 expression nor its subcellular localization were reported following chemotherapy. It should be noted that etoposide induced p53 activity in D283, MEDI and D458 MB cell lines *in vitro* [42] and the p53 target miR-34a was able to reduce the viability in the p53-impared MB cell line, MEB-Med8a [43], however the effects of silencing of p53 *per se* were not reported. Furthermore, docosahexaenoic acid and etoposide were found to reduce the levels of MDM2 in both p53-mutant DAOY cells as well as in p53-wildtype D283 cells [44] and we also observe decreases in MDM2 with VMY, along with rapid translocation of p53 into the nucleus. Collectively these published studies and our new data suggest that components of the p53 pathway remain intact in a variety of p53-mutant and p53-wild type MB cells.

**Figure 8:**
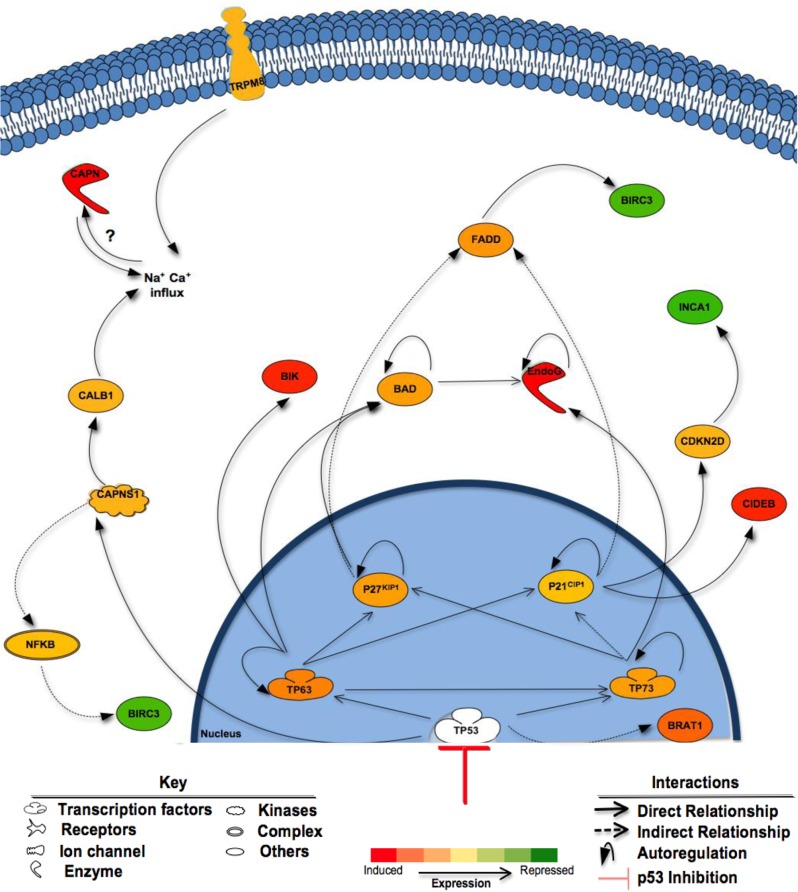
Proposed mechanisms of enhanced cell death following inhibition of p53 Shown are the effects of p53 suppression on components of cell death pathways in Pif + VMY *vs.* VMY treated D556 cells. p53 inhibition by Pifithrin resulted in the induction of *p63* and *p73* genes and subsequent enhanced cell death via apoptosis. Induction of the *p63* and *p73* genes leads to the activation of *p21^1^* and *p27^KIP1^* both of which can indirectly trigger FADD, reducing the expression of BIRC3 (cIAP2). Induction of p73 led to large increases in EndoG and CIDEB expression leading to DNA fragmentation while increased levels of p63 induced apoptosis though BIRC3 and BIK, the latter of which along with TRPM8 can influence intracellular calcium levels. BAD; BCL2-Associated Agonist Of Cell Death, BIK; BCL2-Interacting Killer (Apoptosis-Inducing), BIRC3; baculoviral IAP repeat containing 3 (cIAP2), BRAT1; BRCA1-Associated ATM Activator 1, CAPN; Calpain, CALB1; Calbindin 1, CDKN2D; Cyclin-Dependent Kinase Inhibitor 2D (p19^Ink4D^), CIDEB; Cell Death-Inducing DFFA-Like Effector B, EndoG; Endonuclease G, FADD; Fas-Associated Via Death Domain, INCA1; Inhibitor Of CDK, Cyclin A1 Interacting Protein 1, NF-KappaB; Nuclear Factor Of Kappa Light Polypeptide Gene Enhancer In B-Cells, TRPM8, Transient receptor potential cation channel subfamily M member 8.

It was therefore surprising that rather than causing chemotherapeutic resistance, the suppression of p53 function by either shRNA knockdown or Pif sensitized DAOY and D556 cells to both VMY and doxorubicin. Mechanistically, the induction of the *p63* and *p73* and their targeted genes by VMY in the Pif-treated cells was one of the most prominent features (Table 1 and Figure 8). These p53 family-member genes, and their various splice variants, play both similar and distinct roles in development as well as in cancer (reviewed in [45]) and can interact with each other with a high degree of complexity. There is abundant evidence that modulation of p53 function can influence the activity of p63 and p73 (reviewed in [46]) and conversely that p63 and p73 can influence p53 activity in adult neural precursor cells [47]. While the mechanism(s) by which the genetic knockdown or chemical suppression of p53 regulates *p63* and *p73* expression in MB cells has yet to be elucidated, our data suggest that the induction of *p63* and *p73* following p53 suppression fundamentally alters the pro-apoptotic machinery in MB cells (Fig 8). It is also unknown whether the increased sensitivity seen in the cell lines tested extends to a broader array of clinical samples or to the chemo-radiation interventions currently used for treating MB. However as both DAOY and D556 cells show similar sensitivities to p53 functional blockade, the possibility exists that at least a subset of the p53 mutations found in MB patients may not adversely impact p53-targeting regimens. Additional experiments assessing whether the p53 mutant proteins identified in recurrent MB exhibit similar responses to combined p53 suppression and exposure to VMY, doxorubicin or other drugs are clearly warranted.

## MATERIALS AND METHODS

### Cell lines and cell culture

The human medulloblastoma (MB) cell lines D556 and DAOY were maintained in complete DMEM containing 10% FBS, L-glutamine, and 100 U/ml Penicillin-Streptomycin as previously described [12]. DNA STR fingerprint analyses were performed on both cell lines as a quality control measure. The DAOY data matched the ATCC database for this line, while early and late passage D556 cultures were compared with no significant changes observed and no matches with the available STR database (not shown).

### Cell viability and growth

Cell viability was determined using trypan blue dye exclusion and viable and total cell counting using a hemocytometer as previously described [11, 12, 15].

### Colony forming assays

A total of 1000 cells were plated in 6 well plates. Cells were allowed to adhere for 24 hrs before treatment, at which point they were treated with VMY or Doxorubicin for 18 hrs. The media was changed after 18hrs and the plates were incubated in the absence of drug for 3-5 days to reach 80% confluency in the negative control wells. Cells were washed with PBS, fixed with 10% neutral buffered formalin solution for 15-30 minutes and stained with 0.5% (w/v) crystal violet for 30-60 minutes. The crystal violet was aspirated, cells were washed with PBS and dried for one hour before counting.

### Flow cytometry

The prostate cells were fixed and stained with 20ug/ml propidium iodide (PI) and 5 U RNase A, and the DNA content and subG1 DNA fragmentation was measured using a FACStar Plus system (Becton-Dickson, Franklin Lakes, NJ) as previously described [11, 12]. Cellular apoptosis was also assessed by APC-Annexin V antibody (Biolegend, San Diego, CA) staining immediately after treatment with VMY and analyzed using FACStar Plus dual laser FACSort system (Becton-Dickson, Franklin Lakes, NJ) as previously described by us [11, 12, 48, 49].

### Immunoblotting

Protein extracts were prepared and separated on 4-20% Tris-glycine gels and electroblotted onto PVDF membranes as previously described [11, 12, 50]. Protein levels were assessed using antibodies against p53 (Millipore, Bellerica, MA #05-224), p-ATM (Cell Signaling, Danvers, MA #5883P), p-Chk2 (Cell Signaling, Danvers, MA #2661P), p-Chk1 (Cell Signaling, Danvers, MA #2348P), p38 (Cell Signaling, Danvers, MA #8690), histone γ-H2AX (Cell Signaling, Danvers, MA #7631), p-histone γH2AX (Cell Signaling, Danvers, MA #9718P), p-BRCA1 (Ser1524) (Cell Signaling, Danvers, MA #9009P), p-P38 MAPK (Cell Signaling, Danvers, MA #9216S), mTOR (Cell Signaling, Danvers, MA #2983), p-ATR (Cell Signaling, Danvers, MA #2853P), p-p53 (Cell Signaling, Danvers, MA #9286P), p-MNK1 (cell signaling, #2111S), p-4E-BP1 (Cell Signaling, Danvers, MA #2855S), MDM2 (Santa Cruz Biotechnology, #sc-965), β-actin (Cell Signaling, Danvers, MA #4967). Densitometry was performed using ImageJ analysis software (NIH, Bethesda, MD) as previously described [11, 12, 50].

### Immunofluorescent imaging

Cells were seeded on glass coverslips and treated with DMSO or VMY for 4 or 18 hrs. Cells were washed with PBS and fixed in 10% formalin for 10 min. The coverslips were washed three times with PBS, the cells were permeabilized with 0.1% Triton X-100 and washed three times with PBS. The samples were blocked with 1% BSA for 20 minutes and washed an additional three times in PBS. The cells were exposed to anti-p53 (1:150, Millipore #05-224) or anti-γH2AX (1:150, Cell Signaling #7631) antibodies for 1 hr at room temperature. The slides were washed with PBS an additional three times and stained with the secondary antibody Alexa Fluor goat 488 anti-mouse (1:150, Life Technologies, A-10667) for 30 min at room temperature. Slides were then counter-stained with DAPI for 5 min. The coverslips were mounted onto glass slides with Tris-buffered fluoro-gel (Electron Microscopy Sciences). Confocal microscopy was performed on a Zeiss (Thornwood, NY) LSM510 Meta microscope using a 40x lens.

### LC3-GFP

LC3 translocation was detected using the green fluorescent protein (GFP)-fused LC3 construct that was generously donated by Dr Robert Clarke [51]. Briefly, cells were seeded in 6 well plates containing glass coverslips and allowed to attach overnight. The LC3-GFP expression plasmid (14ug) was transfected using Lipofectamine LTX reagent (Life Technologies, Carlsbad, CA #15338-100) as previously described by us [15]. 24 hours after transfection, the cells were treated with VMY or vehicle. After 18 hours, the coverslips with attached cells were stained with DAPI and rinsed 3 times with PBS and the coverslips mounted. Imaging was performed by confocal microscopy as previously described [12, 15].

### Autophagy inhibitors

For autophagy inhibition, 3-methyladenine (3-MA) (Sigma-Aldrich, St Louis, MO #M921) was used at 5mM and chloroquine diphosphate (CQ) (Sigma-Aldrich, St Louis, MO #C6628) was used at 50 μM as previously described [15]. Cells were exposed to these inhibitors for 20 minutes prior to treatment with either DMSO or VMY [15].

### p53 expression and shRNA knockdown

For lentivirus knockdown experiments, the p53shRNA and pLKO vectors were purchased commercially (Vector Biolabs, Philadelphia, PA, #1854) and used as described by the manufacturer as previously described [15]. Briefly, 293T cells (ATCC, Manassas, VA) were cotransfected with shRNA constructs along with the pHR′8.2ΔR and pCMV-VSV-G helper constructs. After 24 hours, the media was changed and the virus-containing media was harvested after an additional 24 hours of incubation. The MB cells were seeded at 30% confluency and viral infections were performed for 72 hours prior to treatment with VMY or DMSO. Efficiency of the knockdown was monitored by p53 immunoblotting and quantification by ImageJ as previously described [15, 52, 53].

### Chemical inhibition of p53

For chemical inhibition of p53, 30uM Pifithrin-α (Sigma-Aldrich, St Louis, MO #P4359) was added one hour prior to treatment with VMY, doxorubicin or DMSO.

### DNA fragmentation

D556 cells were infected with pLKO or p53shRNA virus's for 72 hrs prior to treatment with VMY or DMSO. Doxorubicin was used as a positive control. The genomic DNA was isolated after 18 hr treatment with VMY or doxorubicin using the DNeasy blood and tissue kit (Qiagen, MD #69506). 500ng of DNA was run on 1% agarose gel containing ethidium bromide with the electrophoresis carried out at 100V for one hour.

### RNAseq and pathway analyses

Total RNA was extracted from D556 cells treated with Pif and VMY as described above using an RNeasy Plus Mini Kit (Qiagen, MD, #74134) and submitted to Otogenetics Corporation (Norcross, GA USA) for RNA-Seq assays. Sequencing was performed on the Illumina HiSeq 2500 (20 million reads, Rapid run, Illumina, CA USA) with chemistry v1.0 and using the 2×106bp paired-end read mode and original chemistry from Illumina according to the manufacturer's instructions. The initial data analysis was started directly on the HiSeq 2500 System during the run. The HiSeq Control Software 2.0.5 in combination with RTA 1.17.20.0 (real time analysis) performed the initial image analysis and base calling. Quality control (QC) was performed using FastQC software. All the samples passed the “Basic Statistics”, “Per Base Sequence Quality”, “Per Sequence Quality Scores”, “Per Base N Content”, and “Sequence Length Distribution”. No specific filtering was done for the samples. The final FASTQ files comprising the sequence information which was used for all subsequent bioinformatics analyses. Sequences were demultiplexed according to the 6bp index code with 1 mismatch allowed. After QC, Tophat2 was used for the alignment, and BAM files were obtained. Partek Genomics Suite (6.6 version 6.12.0713 software (Partek Inc.) was utilized to calculate RPKM as normalization, and fold changes were calculated based on the RPKM results. The pathways analysis was performed through the use of QIAGEN's Ingenuity^®^ Pathway Analysis (Qiagen, Redwood City, CA).

## SUPPLEMENTAL FIGURES


