# Supplementary material for: The p53 tumor suppressor protein protects against chemotherapeutic stress and apoptosis in human medulloblastoma cells
Source: Aging (Albany NY). 2015 Oct 27;7(10):854–67. doi: 10.18632/aging.100831 (PMC4637210; doi:10.18632/aging.100831)
Supplement: Supplementary file 1 [file aging-07-854-s001.pdf]

SUPPLEMENTAL FIGURES

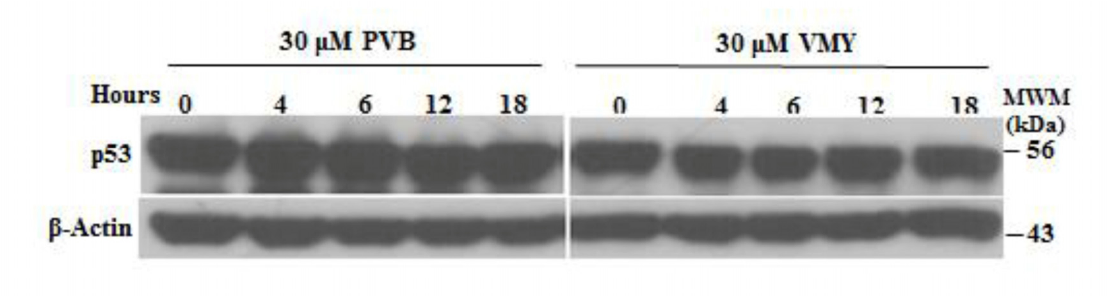

Supplemental Figure S1. Immunoblotting for p53 following exposure of D556 cells to PVB and VMY for the times indicated. b-actin was used as a loading control. MWM (kDa); molecular weigh marker in kilodaltons.

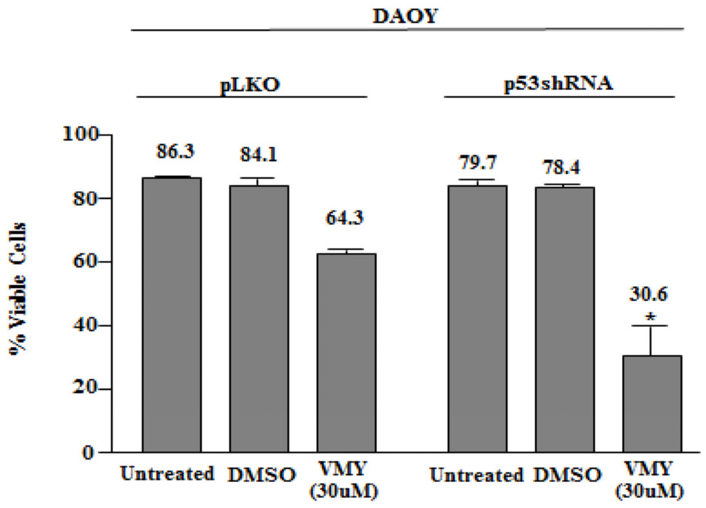

Supplemental Figure S2. Infection of MB cells with pLKO or p53shRNA. Three days after infection, DAOY cells were either left untreated or exposed to DMSO or VMY for 18 hrs. Cell viability was determined by trypan blue dye exclusion. The data are shown as average ± standard deviation of N= 2 separate experiments. *; $p < 0.05$.
